# Hospital-associated decline: A concept analysis

**DOI:** 10.1016/j.ijnsa.2026.100700

**Published:** 2026-10-03

**Authors:** Amanda Brolly, Irene Hartigan, Ruth McCullagh, Teresa Wills, Patrick Cotter

**Affiliations:** a School of Nursing and Midwifery, University College Cork, Ireland; b Discipline of Physiotherapy, School of Clinical Therapies, University College Cork, Ireland; c University Hospital Kerry, Rathass, Tralee, Co. Kerry, V92 NX94, Ireland

**Keywords:** Activities of daily living, Deconditioning, Functional decline, Hospitalization, Mobility, Older people

## Abstract

**Background:**

Hospital-associated decline is commonly described as loss of independence in daily activities yet is increasingly recognised as multidimensional, encompassing physical, cognitive, and psychological domains. However, research has increasingly progressed towards intervention development without a clearly stabilised conceptual foundation, limiting the consistency and comparability of measurement and intervention approaches.

**Objective:**

To clarify the concept of hospital-associated decline to inform its management and measurement in the acute hospital setting.

**Design:**

This concept analysis was conducted using the Walker and Avant framework.

**Data sources:**

Five databases were searched according to predefined eligibility criteria. Observational studies involving inpatients aged ≥65 years admitted to acute medical or geriatric settings were included. Data on attributes, antecedents, consequences, and empirical referents were synthesised to clarify and describe the concept of hospital-associated decline.

**Results:**

Twenty-seven studies were included in the final conceptual synthesis. Hospital-associated decline emerged as a complex, time-defined process occurring within the context of patient vulnerability, acute illness, and hospital care processes, with deterioration in activities of daily living performance representing the defining attribute and consistently measured manifestation. Although researchers increasingly conceptualise hospital-associated decline as multidimensional, it continues to be operationalised predominantly through functional measures, most frequently the Barthel and Katz indices. Cognitive and psychological domains were rarely assessed and inconsistently specified as outcomes. Decline was measured across defined observation periods, typically from premorbid baseline or admission to discharge, with some studies extending follow-up to 6 months. Antecedents were clustered into three primary domains: patient vulnerability, acute illness, and hospital care processes. These hospital care processes included low mobility, inadequate nutrition, restraint use, prolonged hospital stay, aspiration, adverse drug reactions, and low nurse staffing levels. Consequences included adverse functional trajectories, increased healthcare utilisation, and mortality. Overall, we have demonstrated a clear disconnect between the multidimensional conceptualisation of hospital-associated decline and its predominant operationalisation using functional measures.

**Conclusion:**

From these findings, we have extended the understanding of hospital-associated decline beyond functional decline alone to a complex, time-defined process. The Nursing Theory of Complex Adaptive Systems provides a theoretical framework for considering how patient vulnerability, acute illness, and hospital care processes may interact and evolve across the hospital episode, while highlighting the implications of changing patient needs for responsive nursing care and resource allocation.

What is already known

● Hospital-associated decline is typically operationalised as functional decline.

● Measurement commonly compares premorbid or admission function with discharge or post-discharge function.

What this paper adds

● Hospital-associated decline is a time-defined process occurring in association with the hospital episode.

● The Nursing Theory of Complex Adaptive Systems provides a framework for understanding how hospital-associated decline antecedents may interact.

● Hospital-associated decline is conceptualised multidimensionally but measured predominantly through functional decline.

## 1 Introduction

Hospitalisation represents a critical period of vulnerability for older adults (Verdon *et al.*, 2025). Although acute care aims to treat illness and restore health, it frequently coincides with new or accelerated functional decline that is not fully explained by disease severity alone (Covinsky Pierluissi and Johnston, 2011). This phenomenon, commonly termed hospital-associated decline, is a potentially preventable source of harm, with implications for recovery (Zisberg *et al.*, 2015), independence (Hajduk *et al.*, 2020), and health system utilisation (Basic *et al.*, 2017). Older adults who experience hospital-associated decline are more likely to have poorer long-term outcomes following discharge (Dasgupta and Brymer, 2015). Despite increasing clinical and research attention, hospital-associated decline lacks a stable conceptual foundation, resulting in misalignment between how it is defined, measured, and targeted in intervention research (Hao *et al.*, 2026; Buurman *et al.*, 2011).

Hospital-associated decline has traditionally been conceptualised as decline in everyday activities, typically operationalised as activities of daily living, including mobility, self-care, and feeding (Giacomino *et al.*, 2023). However, this functional framing provides only a partial representation of decline during hospitalisation, with cognitive and psychological dimensions inconsistently incorporated into definitions and measurement (Zisberg *et al.,* 2016; Verdon *et al.*, 2025). Definitions, measurement approaches, and observation periods vary substantially across studies, limiting comparability and obscuring the underlying construct (Westlake *et al.*, 2025). This lack of conceptual clarity has important implications for intervention development and evaluation (Chen *et al.*, 2022). Although multicomponent and implementation-informed interventions have advanced, they have often been developed without explicit alignment to a clearly specified construct of hospital-associated decline (Covinsky *et al.*, 2003). As a result, interventions may target only a partial representation of decline, contributing to variability in both intervention design and outcome reporting (Baldwin *et al.*, 2026; Adsett and Mudge, 2024). Without clear specification of factors contributing to hospital-associated decline, it remains difficult to distinguish core intervention components from context-dependent elements, limiting transferability and scalability beyond local settings (Covinsky *et al.*, 2003; Naughton *et al.*, 2024).

Health systems emphasise the preservation of function and person-centred outcomes during healthcare encounters (Conroy and van Oppen, 2023; Adsett and Mudge, 2024). For example, the Age-Friendly Health Systems 4Ms framework provides a multidomain approach to care, focusing on What Matters, Medications, Mind, and Mobility (Health Service Executive and Department of Health, 2025). However, prevailing conceptualisations of hospital-associated decline align most closely with the physical function and Mobility domains (Chenhuichen *et al.*, 2024; Nagae *et al.*, 2022), while Mind, encompassing cognitive and emotional wellbeing, and What Matters are variably incorporated, and Medication-related factors are inconsistently considered (Chen *et al.*, 2022)**.** This highlights the misalignment between multidomain care frameworks and the predominantly-functional operationalisation of hospital-associated decline (Naughton *et al.*, 2024).

In the absence of conceptual clarity, functional decline is often attributed to acute illness severity, obscuring the contribution of hospital environments and care processes as modifiable factors (Zisberg *et al.*, 2015; Nagae *et al.*, 2023). Although researchers have examined risk factors and outcomes associated with hospital-associated decline, few have examined the concept itself. This limits the ability to standardise measurement, evaluate interventions, and translate evidence into practice (Gordon, Grimmer and Barras, 2019). A systematic, empirically-grounded clarification of hospital-associated decline is essential to inform efforts to prevent or mitigate it (Loyd *et al.*, 2020).

To address this gap, a concept analysis of hospital-associated decline grounded in empirical evidence was undertaken. Applying the Walker and Avant (2019) framework, we synthesised findings from observational studies involving adults aged ≥65 years admitted to acute medical or geriatric settings to identify the defining attributes, antecedents, consequences, and empirical referents of hospital-associated decline.

## 2 Methods

Concept analysis provides a systematic approach to clarifying the meaning and boundaries of a concept (Fitzpatrick and McCarthy, 2016). The analysis was conducted using Walker and Avant's (2019) eight-step framework consisting of: (i) selecting the concept, (ii) determining the aims of the analysis, (iii) identifying all uses of the concept, (iv) determining the defining attributes, (v) identifying a model case, (vi) identifying other types of cases, (vii) identifying antecedents and consequences, and (viii) defining empirical referents. Hospital-associated decline was selected as the concept because of variation in its terminology, conceptualisation, and measurement across the literature. The aim of the analysis was to clarify the concept of hospital-associated decline by identifying its defining attributes, antecedents, consequences and empirical referents. Uses of the concept were examined within the literature to establish its conceptual boundaries and are presented in the results. Findings from a structured review of primary studies were synthesised narratively.

### 2.1 Search strategy and selection criteria

The literature search was guided by the Population, Exposure, and Outcome framework, appropriate for concept analysis, given the focus on identifying the aetiology of hospital-associated decline (Hosseini *et al.*, 2024). The population was defined as acute medical or geriatric inpatients (≥ 65 years). The exposure was admission to a medical or geriatric ward in an acute hospital setting. The outcome of interest was functional change across hospital-related time points, including but not limited to physical, cognitive, and psychological measures.

A comprehensive search strategy was developed and trialled in collaboration with a health sciences librarian and refined to ensure methodological rigour. The preliminary findings of the search informed the refinement of search terms. A systematic search was conducted on 4 November 2024 across five electronic databases: CINAHL Plus with Full Text (EBSCO), MEDLINE (Ovid), Scopus, APA PsycINFO, and ProQuest Dissertations and Theses. Keywords and related terms were adapted for each database, and Boolean operators were used (Supplementary Material Table S1). No language restrictions were applied. An updated search was conducted on 22 January 2026 to identify literature published since the original search. The same core search terms and eligibility criteria were applied; however, CINAHL Ultimate was used for the updated CINAHL search. Following screening of the updated search results, one additional study met the eligibility criteria. ProQuest Dissertations and Theses was selected as the primary source of grey literature, consistent with methodological recommendations for concept analysis (Walker and Avant, 2019; Booth, Sutton and Papaioannou, 2016). Finally, the reference lists of relevant articles, including other review articles in the field, were manually searched.

Eligibility criteria were developed iteratively (Supplementary Material Table S2). For example, initial inclusion of whole-hospital populations was subsequently refined to acute medical and geriatric wards to improve conceptual precision in defining hospital-associated decline. Consistent with the aim of identifying empirically observable indicators, inclusion was restricted to quantitative studies reporting measurable outcomes, and qualitative studies were excluded. To ensure relevance to our contemporary acute care system, eligibility was further restricted to studies published in the past 10 years and conducted in countries with a very high Human Development Index (United Nations Development Programme, 2024).

All records identified through database searching, reference list screening, and an updated search were imported into Covidence Systematic Review Software. Following duplicate removal, articles were screened by reviewers independently and in pairs, initially by title and abstract, then by full text. Conflicts were resolved through a third reviewer. A data extraction table was refined iteratively between April and December 2025 through repeated engagement with the literature. Initial extraction fields were informed by established methodology and refined through repeated examination of included studies (Walker and Avant, 2019; Booth, Sutton and Papaioannou, 2016). Data were extracted under the following headings: author, year, country, study design, aim, sample size, key demographics, empirical referents, observation period for decline, attributes, antecedents, and consequences (Walker and Avant, 2019) (Supplementary Material Table S3).

During data extraction, defining attributes and empirical referents were identified from how hospital-associated decline was operationalised and measured across the included studies. Initial extraction revealed substantial heterogeneity in the variables and constructs examined across the studies. Extensive lists of possible antecedents and consequences were extracted, with no clear patterns emerging. Therefore, to manage this heterogeneity, variables demonstrating a statistically-significant independent association with hospital-associated decline in at least one multivariable analysis were retained. Together, these approaches supported the systematic identification of defining attributes, empirical referents, antecedents, and consequences.

## 3 Results

The database search and reference list screening yielded 2,305 records. After removal of duplicates, 1,610 titles and abstracts were screened, and 149 articles were retrieved for full-text review. Of the 149 articles assessed for eligibility, 104 were excluded, most commonly because they fell outside the predefined 10-year publication period (*n* = 65). Forty-five studies met the initial eligibility criteria and were included for data extraction. Following an analytical refinement to retain only studies reporting significant findings from multivariable analyses, 27 studies were included in the final conceptual synthesis. The study selection process is presented in the PRISMA flow diagram (Supplementary Material Figure S1).

### 3.1 Empirical characteristics of included studies

Of the 27 included studies, 19 were cohort studies, with the remainder comprising cross-sectional, case-control, and observational studies. Studies were conducted predominantly in Europe, followed by Asia, North America, and Australia. Sample sizes varied substantially, ranging from 70 to 33,111 participants (median 398, interquartile range 188 to 1,698). Eight studies included ≤ 200 participants, eleven included 201-999 participants, and eight included ≥ 1,000 participants. Detailed characteristics of included studies are provided in Supplementary Material Table S3.

Hospital-associated decline was predominantly assessed using activities of daily living-based instruments, commonly the Barthel Index (*n* = 12, 44.4%), and the Katz Index (*n* = 8, 29.6%), with decline typically defined as a ≥1-point or ≥5-point reduction depending on the instrument version used. A smaller group of studies adopted broader functional disability constructs, such as composite measures and measures of mobility and muscle strength. One large cohort classified decline as an observable increase in assessed nursing care needs between admission and discharge, reflecting a care-need-based, rather than task-specific conceptualisation (Morioka *et al.*, 2025). The Barthel and Katz measurements were applied across regions, with the Barthel Index used more in Southern Europe and parts of Asia, while the Katz Index was used more in North America and Northern Europe. This variation likely reflects convention rather than differences in the underlying construct of decline.

All approaches conceptualised hospital-associated decline as measurable change in functional independence over time, anchored either to pre-admission baseline or to status at hospital entry. Observation periods varied substantially. Most defined decline relative to a premorbid baseline assessed on admission, comparing pre-hospital function with follow-up status, and less often with discharge status. Only two studies measured change occurring during hospitalisation (admission to discharge), thereby capturing in-hospital deterioration only (Morioka *et al.*, 2025; Freeman *et al.*, 2023). Three studies assessed from hospital admission to the post-discharge period (Yoo *et al.*, 2019; Huang *et al.*, 2021; Aarden *et al.*, 2021), identifying persistent or delayed functional recovery. These observation periods are illustrated in Figure 1 with detailed measurement approaches and observational periods summarised in Supplementary Material Table S3.

**Fig. 1:**
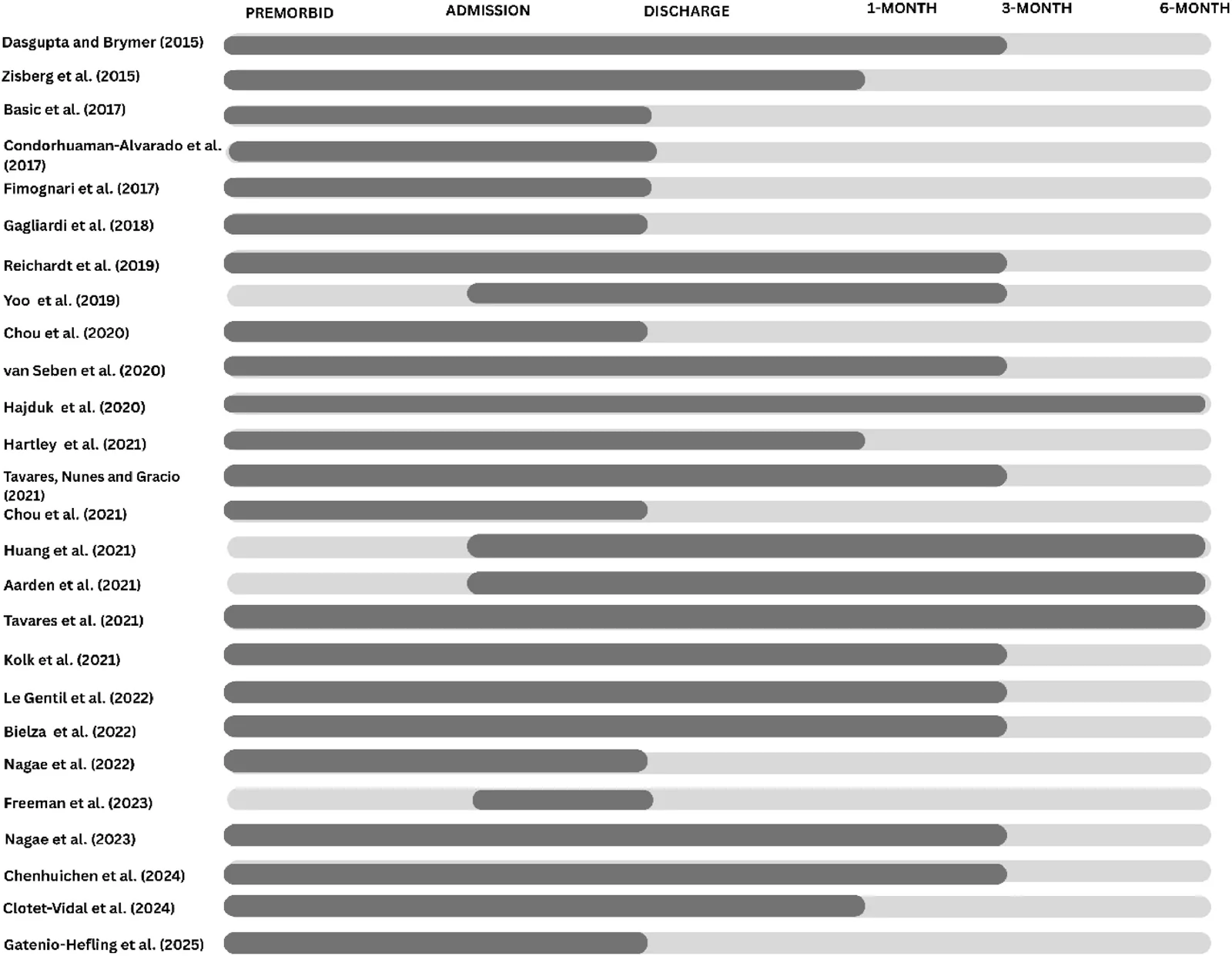
Observation periods and temporal mapping across included studies.

Horizontal bars indicate the earliest and latest assessment points used to identify functional decline, spanning premorbid status, admission, discharge and follow-up, up to 6 months post-discharge. Variation in bar length illustrates heterogeneity in the timing of outcome measurement across studies.

### 3.2 Antecedents of hospital-associated decline

Antecedents are the conditions that must exist before hospital-associated decline can occur (Walker and Avant, 2019). Across included studies, antecedents of hospital-associated decline clustered into three domains: patient-related vulnerability, acute illness and hospital care processes (Table 1). Patient-related antecedents reflected reduced physiological and functional reserve prior to admission. These included advanced age, lower premorbid activities of daily living function, reduced strength and mobility, frailty, pre-existing cognitive impairment, depressive symptoms, and a higher burden of co-morbid conditions. In addition, pre-existing geriatric syndromes — including pressure sores, sensory impairment, falls risk, incontinence and malnutrition — were consistently associated with vulnerability to decline. Hospital-associated decline was also observed among individuals with higher premorbid function and muscle strength, reflecting a greater measurable range for decline (Basic *et al.*, 2017; Hartley *et al.*, 2021). Taken together, these patient-related antecedents reflect reduced physiological and functional reserve at admission, predisposing individuals to disproportionate functional loss during acute illness and hospitalisation (Chou *et al.*, 2021).

**Table 1:** Synthesised antecedents, defining attribute and consequences of hospital-associated decline.

Conceptual component	Synthesised theme	Description	Supporting studies
Antecedents	**Patient vulnerability (premorbid)**	Advanced age; lower premorbid function; frailty; malnutrition; multimorbidity; cognitive impairment; depression; geriatric syndromes (e.g., falls risk, incontinence)	Dasgupta and Brymer, 2015; Zisberg et al., 2015; Condorhuaman-Alvarado et al., 2017; Gagliardi et al., 2018; Yoo et al., 2019; Reichardt et al., 2019; Hajduk et al., 2020; Tavares, Nunes and Grácio, 2021; Tavares et al., 2021; Chou et al., 2021; Hartley et al., 2021; Bielza et al., 2022; Le Gentil et al., 2022; Freeman et al., 2023; Chenhuichen et al., 2024; Clotet–Vidal et al., 2024; Gatenio–Hefling et al., 2025
	**Acute illness**	Delirium; dehydration; additional in-hospital diagnoses (e.g., pneumonia)	Dasgupta and Brymer, 2015; Condorhuaman-Alvarado et al., 2017; Tavares, Nunes and Grácio, 2021; Nagae et al., 2023; Freeman et al., 2023
	**Hospital care processes**	Prolonged hospital stay; low nurse staffing levels; restraint use; low in-hospital mobility; inadequate nutrition; aspiration; adverse drug reactions	Dasgupta and Brymer, 2015; Zisberg et al., 2015; Basic et al., 2017; Condorhuaman-Alvarado et al., 2017; Fimognari et al., 2017; Yoo et al., 2019; Hartley et al., 2021; Chou et al., 2020; Hajduk et al., 2020; Tavares, Nunes and Grácio, 2021; Chou et al., 2021; Le Gentil et al., 2022; Freeman et al., 2023; Chenhuichen et al., 2024; Clotet-Vidal et al., 2024; Morioka et al., 2025
Attribute	**Decline in activities of daily living performance**	Observable reduction in independence in activities of daily living (e.g., feeding, bathing, dressing, continence, transferring, mobility)	Dasgupta and Brymer, 2015; Zisberg et al., 2015; Basic et al., 2017; Condorhuaman-Alvarado et al., 2017; Fimognari et al., 2017; Gagliardi et al., 2018; Yoo et al., 2019; Reichardt et al., 2019; Chou et al., 2020; Hajduk et al., 2020; van Seben et al., 2020; Chou et al., 2021; Tavares, Nunes and Grácio, 2021; Hartley et al., 2021; Huang et al., 2021; Tavares et al., 2021; Kolk et al., 2021; Bielza et al., 2022; Le Gentil et al., 2022; Nagae et al., 2022; Freeman et al., 2023; Nagae et al., 2023; Clotet-Vidal et al., 2024; Chenhuichen et al., 2024; Gatenio-Hefling et al., 2025; Morioka et al., 2025
Consequences	**Persistent functional decline**	New care needs following discharge	Zisberg et al., 2015; Gagliardi et al., 2018; Reichardt et al., 2019; Hajduk et al., 2020; Huang et al., 2021; Hartley et al., 2021; Tavares et al., 2021; Kolk et al., 2021; Bielza et al., 2022; Nagae et al., 2023; Chenhuichen et al., 2024; Gatenio-Hefling et al., 2025; Morioka et al., 2025
	**Institutionalisation**	Increased likelihood of nursing home admission	Tavares, Nunes and Grácio, 2021; Clotet-Vidal et al., 2024
	**Increased healthcare utilisation**	Higher rates of hospital readmission or emergency department attendance following discharge	Dasgupta and Brymer, 2015; Basic et al., 2017; Chou et al., 2020; van Seben et al., 2020; Chou et al., 2021
	**Mortality**	Increased risk of death following hospitalisation	Chou et al., 2020; van Seben et al., 2020

Several studies identified functional decline occurring between premorbid baseline and admission, indicating deterioration before admission. In studies measuring delirium, this was detected on admission or early during hospitalisation and was consistently associated with hospital-associated decline. Dehydration, measured in only one study, and additional in-hospital diagnoses (e.g., pneumonia, heart failure, colitis), further reflect illness-related vulnerability.

Care processes were associated with hospital-associated decline, including longer length of stay, which was measured heterogeneously, precluding the synthesis of a single average value across studies. Low mobility and falls during hospitalisation, restraint use, poor nutrition, and low nurse staffing levels were also associated with hospital-associated decline. Acute complications arising during admission, including aspiration and adverse drug reactions, were also identified as factors related to decline. Collectively, these findings identify potentially modifiable targets for preventing, reducing, or mitigating hospital-associated decline (Verdon *et al.*, 2025). Table 1 summarises the synthesised antecedents, defining attribute, and consequences of hospital-associated decline.

### 3.3 Defining attributes of hospital-associated decline

Defining attributes represent the key dimensions by which a concept is recognised (Walker and Avant, 2019). Across included studies, reduced performance in basic activities of daily living consistently emerged as the sole defining attribute of hospital-associated decline, with 96% of studies (*n* = 26) operationalising hospital-associated decline in this way.

### 3.4 Consequences of hospital-associated decline

Consequences are outcomes arising from the concept (Walker and Avant, 2019). Although functional decline represents the defining attribute of hospital-associated decline, its persistence at discharge or during post-discharge follow-up was also identified as a consequence. Hospital-associated decline was consistently associated with adverse patient outcomes beyond the acute hospital episode. In 44% of studies (*n* = 12), functional decline was evident at discharge or post-discharge follow-up, with evidence of functional improvement following hospitalisation observed in two studies (*n* = 2).

Patient-level consequences included readmission, mortality and institutionalisation. Length of stay was associated with hospital-associated decline in two studies (*n* = 2); however, as both were measured within the same admission, temporal directionality cannot be established. These findings demonstrate the clinical and operational significance of hospital-associated decline, with consequences extending beyond the acute hospital episode (Adsett and Mudge, 2024; Verdon *et al.*, 2025).

### 3.5 Use of the concept

In hospital settings, decline is often equated with acute clinical deterioration. While early warning systems, such as the National Early Warning Score (Badriyah *et al.*, 2014), are well established for identifying acute clinical deterioration, a more insidious and often overlooked form of decline (functional loss) occurs among hospitalised patients (Loyd *et al.*, 2020). This affects physical, cognitive, and psychological domains (Welch *et al.*, 2024). Although not typically life-threatening, it can result in substantial functional loss, including new mobility limitations and increased dependency after hospitalisation (Zisberg *et al.*, 2015). These observations underpin the development of hospital-associated decline, which has been interpreted differently across studies.

Across the included studies, definitions of hospital-associated decline (Table 2) reflect conceptual evolution and heterogeneity in how decline is observed and measured, with many definitions emphasising loss of independence in basic activities of daily living, despite the broader multidimensional nature of decline. A dominant feature is the characterisation of hospital-associated decline as post-hospitalisation loss of functional independence. This function-based conceptualisation distinguishes hospital-associated change from pre-existing functional disability and remains the primary empirical referent used to identify hospital-associated decline.

**Table 2:** Explicit definitions of hospital-associated decline identified in the literature.

Defining features synthesised across studies	Studies providing an explicit definition
Loss of independence in activities of daily living	Yoo et al., 2019; Hajduk et al., 2020; Tavares, Nunes and Grácio, 2021; Morioka et al., 2025.
Decline is conceptualised as measurable change in function over time	Condorhuaman-Alvarado et al., 2017; Fimognari et al., 2017; van Seben et al., 2020; Chou et al., 2021; Freeman et al., 2023.
Multidimensional decline (physical, cognitive and psychological) *	Naughton et al., 2024; Welch et al., 2024; Arora, 2022.

* Definitions derived from expert opinion or narrative commentary.

Studies were included in this table only when an explicit definition of hospital-associated decline (or closely related construct) was provided. Studies defining decline solely using measurement thresholds (e.g., a change in Barthel or Katz Index) are discussed in the text.

A second recurring feature across definitions was the conceptualisation of decline as change over time. While not all definitions explicitly specified temporal reference points, those that did consistently framed decline as occurring between premorbid function and discharge. Other studies used broader observation periods, assessing change from premorbid baseline to admission, admission to discharge, or into post-discharge follow-up, as illustrated in Figure 1, highlighting variability in the timing of outcome measurement across studies. Overall, hospital-associated decline was conceptualised as a dynamic, time-defined change in functional independence, most often occurring between premorbid baseline and discharge or follow-up, rather than confined to in-hospital deterioration alone. Collectively, these findings demonstrate heterogeneity in how hospital-associated decline is defined and measured.

### 3.6 Proposed definition

Synthesising defining attributes, antecedents, consequences, and empirical referents, the following empirically grounded definition of hospital-associated decline is proposed, reflecting its current operationalisation in the literature.

*Hospital-associated decline is a complex, time-defined process occurring in the context of patient vulnerability, acute illness, and hospital care processes, manifested by measurable deterioration in the performance of activities of daily living.*

### 3.7 Model cases

Model cases illustrate the defining attributes of hospital-associated decline and support conceptual clarification (Walker and Avant, 2019). As decline in activities of daily living performance was the only consistent defining attribute identified in this analysis, a borderline case containing some but not all defining attributes could not be constructed without treating antecedents or individual components of functional measures as additional defining attributes. Consistent with the conceptualisation of hospital-associated decline as a dynamic and measurable process, the included cases demonstrate change in functional status across hospital-related time points, from premorbid baseline through admission and discharge. Detailed case narratives are presented in Supplementary Material File S4.

### 3.8 Empirical referents of hospital-associated decline

Empirical referents are observable indicators of the concept (Walker and Avant, 2019). Across studies, these comprised measurable reductions in functional ability, particularly loss of independence in activities of daily living.

Hospital-associated decline was typically operationalised as a decline in functional performance, most frequently measured using the Barthel Index or Katz Index (Hartigan, 2007; Giacomino *et al.*, 2023). However, definitions varied, with thresholds ranging from a decline of ≥1 point to a decline of ≥5 points (Supplementary Material Table S3), with equivalence between point changes inconsistently specified (Giacomino *et al.*, 2023). Although these measures capture functional status, they do not reflect broader dimensions such as cognition, mood, or hospital processes, which were not consistently operationalised or measured across included studies (Chen *et al.*, 2022).

## 4 Discussion

Recent literature conceptualises hospital-associated decline as a multidimensional construct encompassing physical, cognitive, and psychological domains (Welch *et al.*, 2024; Naughton *et al.*, 2024; Arora, 2022). However, this multidimensional framing is largely described in expert opinion and secondary syntheses rather than operationalised as an empirical outcome (Welch *et al.*, 2024). Consistent with this distinction, in the present analysis, we identified cognitive impairment and depression as antecedents of hospital-associated decline, while the defining attribute remained reduced performance in activities of daily living. This suggests that multidimensional vulnerability forms part of the context in which hospital-associated decline develops, whereas its empirical operationalisation remains predominantly functional, leaving the relationship between cognitive and psychological decline and hospital-associated decline insufficiently examined (Chen *et al.,* 2022; Reichardt *et al.,* 2019). In empirical studies, hospital-associated decline has been characterised either as a discrete functional outcome at discharge (Nagae *et al.*, 2022; Gatenio-Hefling *et al.*, 2025) or a dynamic process unfolding over defined hospital-related time points (Morioka *et al.*, 2025; van Seben *et al.*, 2020). Where functional decline is assessed only at discharge or follow-up, its evolution during hospitalisation may be obscured, limiting understanding of when decline emerges and potentially overlooking opportunities for early identification and intervention (Gordon, Grimmer and Barras, 2019).

Taken together, these findings highlight the importance of considering when functional decline occurs across the hospitalisation episode. This concept analysis advances current understanding by positioning hospital-associated decline as a time-defined process, currently identified through functional decline, occurring in temporal association with the hospitalisation episode, rather than a discrete discharge-based outcome. Figure 1 illustrates that the observation periods used to identify hospital-associated decline span three distinct temporal contexts: pre-hospital, hospital, and post-hospitalisation. Decline occurring between premorbid baseline and admission may predominantly reflect pre-existing vulnerability and the effects of acute illness before exposure to hospital care, whereas change from admission to discharge is more directly temporally associated with the hospitalisation episode, although the respective contributions of patient vulnerability, acute illness, and hospital care processes cannot be disentangled. Post-discharge trajectories may additionally be influenced by recovery, rehabilitation, and social supports (Tavares *et al.,* 2021).

Importantly, these temporal contexts should not be interpreted as necessarily representing equivalent trajectories of decline. Rather, the variation in temporal contexts across studies highlights important conceptual heterogeneity. The evidence does not support identification of a single point at which the “hospital-associated” component begins, nor does it permit causal attribution of decline specifically to hospital care. Accordingly, *hospital-associated* is interpreted here as indicating temporal and clinical association over the duration of the hospitalisation episode rather than direct causal attribution to hospital care. However, the identification of potentially modifiable hospital care processes associated with hospital-associated decline suggests that aspects of care delivery may represent important targets for intervention. Within this temporal framework, the antecedents identified in the concept analysis provide further insight into the factors associated with the development of hospital-associated decline.

The identified antecedents comprise patient vulnerability, acute illness, and hospital care processes. While these findings do not establish interaction between these domains, their co-occurrence provides a basis for considering their potential interaction. Emerging European nursing complexity literature associates greater patient vulnerability with increasing care needs and greater nursing complexity, including needs related to mobility, activities of daily living, and nutrition (Cocchieri *et al.*, 2026). This provides a potential conceptual link between patient vulnerability and hospital care processes, whereby increasing vulnerability may be associated with greater and more complex nursing care requirements. Building on principles of complex adaptive systems (Dooley, 1997), the Nursing Theory of Complex Adaptive Systems frames patient care as arising through dynamic interdependent relationships between patient, nurse and the wider care environment (Notarnicola *et al.,* 2024). Healthcare systems comprise multiple interconnected components whose relationships change over time, such that outcomes cannot necessarily be understood by considering individual components in isolation (Plsek and Greenhalgh, 2001). Applied to hospital-associated decline, this provides a basis for considering how changes in patient vulnerability, acute illness and hospital care processes may combine and evolve across the hospital episode.

Viewed through the Nursing Theory of Complex Adaptive Systems, these findings suggest that nursing assessment should extend beyond identifying individual risk factors to recognising changes in patterns and the accumulation of patient needs across hospitalisation (Plsek and Greenhalgh, 2001). Within this perspective, nursing complexity reflects not simply the number of individual care needs but also the changing demands created as multiple patient needs and care requirements accumulate across the hospital episode (Notarnicola *et al.,* 2024). Rather than addressing these factors as discrete nursing problems, an integrated and responsive multidisciplinary approach, triggered by nurses' recognition of changing patient needs, may be required to preserve function during hospitalisation (Notarnicola *et al.,* 2024; de Foubert *et al.*, 2021). Consequently, the nursing resources required to meet these needs may vary according to patient acuity and dependency (Drennan *et al.,* 2018), suggesting a potential relationship between patient vulnerability, nurse staffing levels and hospital-associated decline. Morioka *et al.* (2025) provide empirical support for the staffing component of this proposed relationship, reporting that nurse staffing shortfalls were associated with an increased risk of hospital-associated decline among hospitalised older adults.

Vulnerability to hospital-associated decline may be reflected in accumulating nursing care needs, potentially providing an opportunity for nurses to recognise increasing vulnerability as patient needs evolve during hospitalisation. Although emerging evidence links frailty-related vulnerability with nursing complexity (Cocchieri *et al*., 2026), how these factors interact with the development of hospital-associated decline remains unclear. Researchers should examine this relationship and its implications for early identification and nursing care.

As several indicators of vulnerability to hospital-associated decline are captured within routine nursing assessment (Chew *et al.,* 2023), researchers should explore whether integration of these data within electronic health records could support recognition of accumulating nursing complexity and risk of functional decline without adding to assessment burden. Earlier recognition, however, is clinically meaningful only if it informs a responsive approach to care that addresses the multiple and changing factors associated with hospital-associated decline (Naughton *et al*., 2024).

This need for a multidomain response is increasingly reflected in intervention research and is consistent with evidence that hospital-associated decline is associated with both individual vulnerability and in-hospital care processes (Zisberg *et al.*, 2015). The field has progressed to multidomain intervention development but without a clear or consistent framework for how multidimensional hospital-associated decline should be defined, measured, and temporally assessed (Westlake *et al.,* 2025). Consequently, interventions vary in both target domains and reported outcomes (Adsett and Mudge, 2024; de Foubert *et al.*, 2021). Although such key components as early mobilisation and multidisciplinary care are well recognised, their implementation remains inconsistent in practice (Verdon *et al.*, 2025; Martínez-Velilla and Gordon, 2025).

We have highlighted the need for alignment between conceptualisation, measurement approach, and assessment timing in relation to the hospitalisation episode (Loyd *et al.*, 2020). In practical terms, longitudinal assessment of hospital-associated decline across the hospitalisation episode and follow-up may better capture its temporal evolution (Chen *et al.,* 2022). Establishing functional status early in the hospital episode may provide a baseline against which subsequent change can be recognised, while longitudinal functional assessment, considered alongside cognitive and psychological assessment, may support recognition of accumulating care needs and increasing complexity (Covinsky Pierluissi and Johnston, 2011; Chen *et al.,* 2022). From a complex systems perspective, this may support recognition of emerging patterns of change and flexible responses to evolving patient needs rather than reliance on predetermined, linear approaches (Plsek and Greenhalgh, 2001). However, implementation of longitudinal assessment within routine care presents practical challenges, including time constraints, workflow integration, and competing clinical priorities (Byrnes McRae and Mudge, 2023; Verdon *et al.*, 2025; Morioka *et al.*, 2025).

Together, these considerations support moving beyond assessment of functional decline alone to considering antecedent risk and in-hospital care processes within a broader multidomain assessment (Hoogerduijn *et al.*, 2010; Tavares, Nunes and Grácio, 2021). Embedding this within existing frameworks, such as the 4Ms (What Matters, Medications, Mind, and Mobility), provides a structured approach to integrating multidomain assessment into routine care (Health Service Executive and Department of Health, 2025).

### 4.1 Limitations

Consistent with the aim of concept analysis to identify defining attributes, antecedents, and consequences, we focused on quantitative studies providing empirically observable and measurable indicators of the concept. Qualitative studies were, therefore, excluded, recognising that this represents a focused rather than an exhaustive conceptual approach. Consequently, dimensions of hospital-associated decline that are more readily captured through patient or healthcare professional experience may not have been fully represented.

To enhance conceptual clarity, the analysis was restricted to variables demonstrating statistical significance in multivariable analyses. While this approach was used to manage heterogeneity within the quantitative literature and prioritise independent associations, the application of a statistical threshold introduced a quantitative logic to the synthesis and may have excluded potentially relevant factors, particularly where statistical significance was influenced by sample size, covariate selection, or analytical models (Polit and Beck, 2008). The antecedents and consequences identified should, therefore, be interpreted as those supported within included quantitative literature, rather than an exhaustive representation of all factors potentially associated with hospital-associated decline. Nevertheless, the resulting domains are broadly consistent with the wider literature, providing convergent support for the conceptual structure identified (Geyskens *et al.,* 2022; Welch *et al.,* 2024).

Furthermore, this analysis was constrained by how hospital-associated decline is operationalised in the literature, where outcomes are largely defined using activities of daily living (Loyd *et al.*, 2020). Consequently, cognitive, psychological, and environmental dimensions of decline — although acknowledged — are under-represented in empirical measurement. This reflects prevailing measurement practices within the field, predominantly based on functional indicators, rather than an intentional simplification within this analysis. Restriction to observational studies in very high Human Development Index countries and to acute medical and geriatric populations, with the exclusion of stroke, oncology, orthopaedic, and surgical populations, limits the generalisability of the proposed concept beyond these clinical settings. Variation in observation periods further complicates conceptual boundaries and cross-study comparisons, as studies may capture different phases of functional decline and recovery across the hospital episode and following discharge.

As a secondary analysis, this work reflects prevailing conceptual trends rather than individual trajectories. While these restrictions limit generalisability, they enabled a more precise delineation of the antecedents and consequences of hospital-associated decline, thereby strengthening conceptual clarity**.** The quantitative focus may, however, have favoured the identification of a defining attribute that is readily measurable, while other dimensions of the concept may be less well represented. Future qualitative and mixed methods research could, therefore, complement these findings and further elucidate dimensions of the concept not adequately captured by existing quantitative measures.

## 5 Conclusion

In this concept analysis, we defined hospital-associated decline as a complex, time-defined process occurring in the context of patient vulnerability, acute illness, and hospital care processes, manifested by measurable deterioration in the performance of activities of daily living. Although hospital-associated decline is predominantly operationalised through functional measures, we have demonstrated that it occurs within a broader, multidimensional clinical context. Viewing functional change across defined hospital-related time points highlights the temporal nature of the concept and identifies potentially modifiable hospital care processes associated with decline. The Nursing Theory of Complex Adaptive Systems provides a framework for considering how patient vulnerability, acute illness, and hospital care processes may dynamically interrelate, while highlighting the implications of changing patient needs for responsive nursing care and resource allocation. Advancing the field requires greater alignment between how hospital-associated decline is conceptualised, when and what is measured, and what is targeted in intervention research.

## Declaration of generative AI and AI-assisted technologies in the manuscript preparation process

During the preparation of this work, the authors used ChatGPT (OpenAI) to support language refinement and to assist in brainstorming and structuring ideas. The authors reviewed and edited all content and take full responsibility for the final manuscript.

## Funding

The research was undertaken as part of a PhD programme of study supported by an employment-based scholarship funded by the Health Service Executive at the College of Medicine and Health, University College Cork.

## CRediT authorship contribution statement

**Amanda Brolly:** Writing – review & editing, Writing – original draft, Conceptualization. **Irene Hartigan:** Supervision. **Ruth McCullagh:** Supervision. **Teresa Wills:** Supervision. **Patrick Cotter:** Supervision.

## Declaration of competing interest

The authors declare that they have no known competing financial interests or personal relationships that could have appeared to influence the work reported in this paper.
