# Supplementary material for: Hospital-associated decline: A concept analysis
Source: Int J Nurs Stud Adv. 2026 Oct 3;11:100700. doi: 10.1016/j.ijnsa.2026.100700 (PMC13683459; doi:10.1016/j.ijnsa.2026.100700)
Supplement: Supplementary file 1 [file mmc1.pdf]

S1 Figure PRISMA

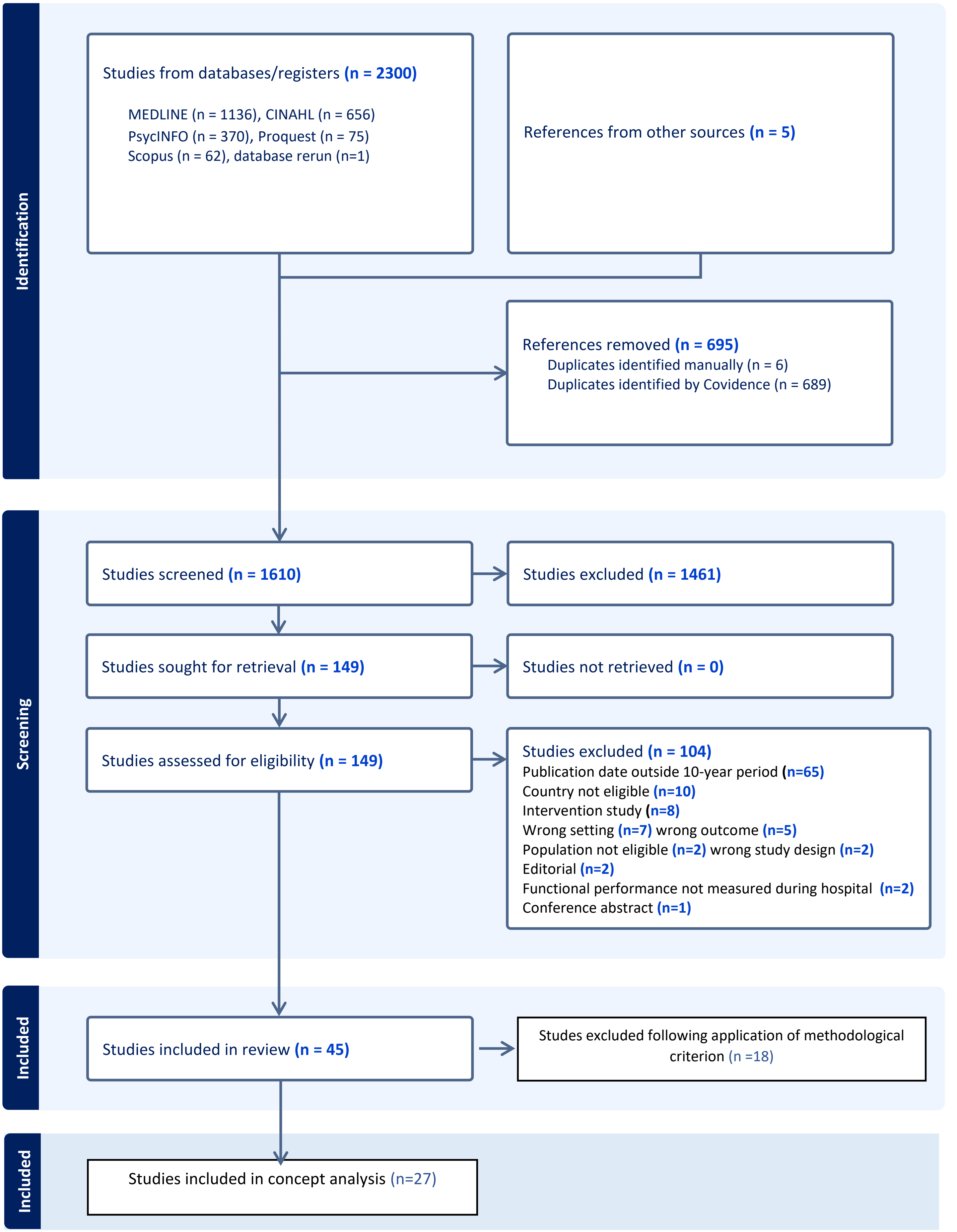

24th April 2026
