# Supplementary material for: Hospital-associated decline: A concept analysis
Source: Int J Nurs Stud Adv. 2026 Oct 3;11:100700. doi: 10.1016/j.ijnsa.2026.100700 (PMC13683459; doi:10.1016/j.ijnsa.2026.100700)
Supplement: Supplementary file 2 [file mmc2.docx]

| P: Older person; E: Hospital O: Functional or cognitive decline | | | | |
| --- | --- | --- | --- | --- |
| Filter: Peer reviewed | | | | |
| Data  base | Justification | Results  04/11/2024 | Updated search period: 04/11/2024 -  22/01/2026 | Search strategy - search terms were initially run independently and then combined into a single search string. |
| CINAHL with full text (EBSCO)  Search was conducted on 04/11/2024 | Extensive coverage of nursing and allied health peer reviewed journals. | 664 | 42  CINAHL Utimate | ((((TI (hospital*) OR AB (hospital*))) OR ((TI ("acute care") OR AB ("acute care"))) OR ((TI (inpatient*) OR AB (inpatient*))) OR ((TI ("acute ward") OR AB ("acute ward"))))) AND ((((TI ("physical performance") OR AB ("physical performance"))) OR ((TI ("behavio?r function") OR AB ("behavio?r function"))) OR ((TI ("mental function") OR AB ("mental function"))) OR ((TI (deteriorat*) OR AB (deteriorat*))) OR ((TI (decondition*) OR AB (decondition*))) OR ((TI ("functional decline") OR AB ("functional decline"))) OR ((TI ("cognitive decline") OR AB ("cognitive decline"))) OR ((TI ("frailty syndrome") OR AB ("frailty syndrome"))) OR ((TI ("cognitive frailty") OR AB ("cognitive frailty"))) OR ((TI ("social frailty") OR AB ("social frailty"))) OR ((TI ("hospital-associated disability") OR AB ("hospital-associated disability"))) OR ((TI ("hospital-associated deconditioning") OR AB ("hospital-associated deconditioning"))) OR ((TI ("post hospital syndrome") OR AB ("post hospital syndrome"))) OR ((TI ("deconditioning syndrome") OR AB ("deconditioning syndrome"))) OR ((TI ("hospital associated functional decline") OR AB ("hospital associated functional decline"))) OR ((TI ("hospital associated decline") OR AB ("hospital associated decline"))) OR ((TI ("adult disuse syndrome") OR AB ("adult disuse syndrome"))) OR ((TI ("HAFD") OR AB ("HAFD"))))) AND ((((TI ("geriatric") OR AB ("geriatric"))) OR ((TI ("frail elderly") OR AB ("frail elderly"))) OR ((TI ("older person") OR AB ("older person"))) OR ((TI ("older adult") OR AB ("older adult"))))) |
| APA PsychInfo  Search was conducted 04/11/2024 | Extensive coverage of articles, books, and dissertations on psychological, social, behavioral and mental health aspects of Hospital-Associated Decline. | 370 | 15 | ((((TI (hospital*) OR AB (hospital*))) OR ((TI ("acute care") OR AB ("acute care"))) OR ((TI (inpatient*) OR AB (inpatient*))) OR ((TI ("acute ward") OR AB ("acute ward"))))) AND ((((TI ("physical performance") OR AB ("physical performance"))) OR ((TI ("behavio?r function") OR AB ("behavio?r function"))) OR ((TI ("mental function") OR AB ("mental function"))) OR ((TI (deteriorat*) OR AB (deteriorat*))) OR ((TI (decondition*) OR AB (decondition*))) OR ((TI ("functional decline") OR AB ("functional decline"))) OR ((TI ("cognitive decline") OR AB ("cognitive decline"))) OR ((TI ("frailty syndrome") OR AB ("frailty syndrome"))) OR ((TI ("cognitive frailty") OR AB ("cognitive frailty"))) OR ((TI ("social frailty") OR AB ("social frailty"))) OR ((TI ("hospital associated disability") OR AB ("hospital associated disability"))) OR ((TI ("hospital associated deconditioning") OR AB ("hospital associated deconditioning"))) OR ((TI ("post hospital syndrome") OR AB ("post hospital syndrome"))) OR ((TI ("deconditioning syndrome") OR AB ("deconditioning syndrome"))) OR ((TI ("hospital associated functional decline") OR AB ("hospital associated functional decline"))) OR ((TI ("hospital associated decline") OR AB ("hospital associated decline"))) OR ((TI ("adult disuse syndrome") OR AB ("adult disuse syndrome"))) OR ((TI ("HAFD") OR AB ("HAFD"))))) AND ((((TI ("older adult") OR AB ("older adult"))) OR ((TI ("older person") OR AB ("older person"))) OR ((TI ("frail elderly") OR AB ("frail elderly"))) OR ((TI ("geriatric") OR AB ("geriatric"))))) |
| Scopus  Search was conducted 04/11/2024 | Largest multi-disciplinary database covering medicine and sciences. | 62 | 427 | (((TITLE("older adult") AND ABS("older adult"))) OR ((TITLE("older person") AND ABS("older person"))) OR ((TITLE("frail elderly") AND  ABS("frail elderly"))) OR ((TITLE(geriatric) AND ABS(geriatric)))) AND (((TITLE("HAFD") AND ABS("HAFD"))) OR ((TITLE("adult disuse syndrome") AND ABS("adult disuse syndrome"))) OR ((TITLE("hospital associated decline") AND ABS("hospital associated decline"))) OR ((TITLE("hospital associated functional decline") AND ABS("hospital associated functional decline"))) OR ((TITLE("deconditioning syndrome") AND ABS("deconditioning syndrome"))) OR ((TITLE("post hospital syndrome") AND ABS("post hospital syndrome"))) OR ((TITLE("hospital associated deconditioning") AND ABS("hospital associated deconditioning"))) OR ((TITLE("hospital associated disability") AND ABS("hospital associated disability"))) OR ((TITLE("social frailty") AND ABS("social frailty"))) OR ((TITLE("cognitive frailty") AND ABS("cognitive frailty"))) OR ((TITLE("frailty syndrome") AND ABS("frailty syndrome"))) OR ((TITLE("cognitive decline") AND ABS("cognitive decline"))) OR ((TITLE("functional decline") AND ABS("functional decline"))) OR ((TITLE(decondition*) AND ABS(decondition*))) OR ((TITLE(deteriorat*) AND ABS(deteriorat*))) OR ((TITLE("mental function") AND ABS("mental function"))) OR ((TITLE("behavio?r function") AND ABS("behavio?r function"))) OR ((TITLE("physical performance") AND ABS("physical performance")))) AND (((TITLE("acute ward") AND ABS("acute ward"))) OR ((TITLE(inpatient*) AND ABS(inpatient*))) OR ((TITLE("acute care") AND ABS("acute care"))) OR ((TITLE(hospital*) AND ABS(hospital*)))) |
| Medline Ovid  Search was conducted 04/11/2024 | Large biomedical database, that includes PubMed, for healthcare and research | 1138 | 43 | ((MH "Hospitals") OR (TI "acute care" OR AB "acute care") OR (MH "Inpatients") OR (TI "acute ward" OR AB "acute ward")) AND  ((MH "Physical Fitness") OR (MH "Motor Activity") OR (TI perform* OR AB perform*) OR ("function") OR (TI "function" OR AB "function")) AND  ((TI "cognitive frailty" OR AB "cognitive frailty") OR (TI "MM Frailty Syndrome") OR (MH "Frailty") OR  (TI "functional decline" OR AB "functional decline") OR (TI "cognitive decline" OR AB "cognitive decline") OR  (TI declin* OR AB declin*) OR (TI deteriorat* OR AB deteriorat*) OR  (TI "hospital associated functional decline" OR AB "hospital associated functional decline") OR  (TI "hospital associated disability" OR AB "hospital associated disability") OR  (TI "post hospital syndrome" OR AB "post hospital syndrome") OR  (TI "adult disuse syndrome" OR AB "adult disuse syndrome") OR  (TI "HAFD" OR AB "HAFD")) AND  ((TI "older adult" OR AB "older adult") OR (TI "older person" OR AB "older person") OR  (TI "frail elderly" OR AB "frail elderly") OR (TI geriatric OR AB geriatric)) |
| ProQuest Dissertations and Theses A&I  Search was conducted 04/11/2024 | Specialist database of grey literature for theses, dissertations and government reports.  (Narrower scope to Google Scholar but more precision and reproducibility. | 75 | 4 | ((abstract(hospital*) OR title(hospital*)) OR (abstract("acute care") OR title("acute care")) OR (abstract(inpatient*) OR title(inpatient*)) OR (abstract("acute ward") OR title("acute ward"))) AND ((abstract("physical performance") OR title("physical performance")) OR (abstract("behavio?r function") OR title("behavio?r function")) OR (abstract("mental function") OR title("mental function")) OR (abstract(deteriorat*) OR title(deteriorat*)) OR (abstract(decondition*) OR title(decondition*)) OR (abstract("functional decline") OR title("functional decline")) OR (abstract("cognitive decline") OR title("cognitive decline")) OR (abstract("frailty syndrome") OR title("frailty syndrome")) OR (abstract("cognitive frailty") OR title("cognitive frailty")) OR (abstract("social frailty") OR title("social frailty")) OR (abstract("hospital associated disability") OR title("hospital associated disability")) OR (abstract("hospital associated deconditioning") OR title("hospital associated deconditioning")) OR (abstract("post hospital syndrome") OR title("post hospital syndrome")) OR (abstract("deconditioning syndrome") OR title("deconditioning syndrome")) OR (abstract("hospital associated functional decline") OR title("hospital associated functional decline")) OR (abstract("hospital associated decline") OR title("hospital associated decline")) OR (abstract("adult disuse syndrome") OR title("adult disuse syndrome")) OR (abstract("HAFD") OR title("HAFD"))) AND ((abstract("older adult") OR title("older adult")) OR (abstract("older person") OR title("older person")) OR (abstract("frail elderly") OR title("frail elderly")) OR (abstract(geriatric) OR title(geriatric))) |
| 3 articles from reference list,  2 recently published scoping reviews |  | 5 |  |  |
