# Supplementary material for: Hospital-associated decline: A concept analysis
Source: Int J Nurs Stud Adv. 2026 Oct 3;11:100700. doi: 10.1016/j.ijnsa.2026.100700 (PMC13683459; doi:10.1016/j.ijnsa.2026.100700)
Supplement: Supplementary file 3 [file mmc3.docx]

**Supplementary Material Table S2. Inclusion and Exclusion Criteria**

| **Characteristic** | **Inclusion criteria** | **Exclusion criteria** |
| --- | --- | --- |
| **Population** | ≥65 years | - $<$65 years - Children - Oncology, palliative care, stroke, surgical and orthopaedic populations, and patients admitted with a primary mental health condition |
| **Exposure** | - Admitted to an acute medical or geriatric ward - Studies including mixed medical and surgical populations were eligible only when data for medical patients were reported or analysed separately | - ICU, high dependency, stroke unit, inpatient mental health unit, emergency department, surgical wards or outpatient settings |
| **Outcome** | - Functional or cognitive decline occurring across hospital-related timepoints. - Decline occurring outside the hospitalisation episode | - Decline not attributable to hospitalisation |
| **Types of studies** | - Observational study designs (cohort, case-control, cross-sectional) | - Reviews and intervention studies (randomised controlled trials) - Qualitative-only studies, case reports, editorials, and opinion pieces |
