# Supplementary material for: Hospital-associated decline: A concept analysis
Source: Int J Nurs Stud Adv. 2026 Oct 3;11:100700. doi: 10.1016/j.ijnsa.2026.100700 (PMC13683459; doi:10.1016/j.ijnsa.2026.100700)
Supplement: Supplementary file 4 [file mmc4.docx]

| 1. Author  2. Year  3. Country | 1. Design  2. Aim | 1. Sample size (N)  2.Key  demographics | 1. Empirical referent (and measurement tools used)  2. Observation period for decline | Antecedents | Attributes | Consequences |
| --- | --- | --- | --- | --- | --- | --- |
| 1. Dasgupta and Brymer  2. 2015  3. Canada | 1. Cohort Study  2. “*To determine how often delirious individuals have certain geriatric syndromes, care issues or additional diagnoses made (after admission), and whether these are associated with poor functional recovery ' pg 794* | 1.N=343 consecutive delirious medical inpatients  2. Mean age 84.5 (≥70 years)  58% female | 1.**Empirical referent**  Functional decline: loss of ability to perform ADLs (OARS Questionnaire)  2.**Observation period**  PM to FU (1 month) | - Older age - Additional diagnosis made after admission - ADL decline before admission - Aspiration - Greater delirium severity - Acute renal failure on admission | *Decline in Activities of Daily Living (ADL) performance* | - Falls since discharge - Repeat hospitalisations - Repeat visits to the emergency department |
| 1. Zisberg et al.  2. 2015  3. Israel | 1. Prospective Cohort Study  2. “*To investigate the combined contribution of processes of hospitalisation and preadmission individual risk factors in explaining functional decline at discharge and 1-month follow-up in older adults with nondisabling conditions*” pg 55 | 1.N= 684 older adults admitted urgently to an acute medical ward in two centres  2. Mean age 78.9 +-5.8yrs  (≥70 years)  50.6% female | 1.**Empirical referents**  Functional decline: >1 point decrease in ADL performance measured using the Modified Barthel Index (MBI) (11 items; score range 0-20)  2.**Observation period**  PM to DC to FU  (3 months) | - Nutritional consumption in hospital - Low in-hospital mobility - Functional decline at discharge - New or increased requirement for continence care in hospital - A longer hospital stay - Lower premorbid ADL function | *Decline in ADL performance* | Functional decline at DC and one month FU |
| 1. Basic, D., Ni Chroinin, D. and Conforti, D.  (2017)  2. 2017  3. Australia | 1. Prospective Observational Study  2*.” To investigate the incidence of, and factors associated with, in-hospital functional decline among older acute hospital patient*s” pg 57 | 1.N = 1693 consecutive older adults admitted to an acute geriatric unit over 5 years  2. Mean age 81.9 years (SD, 7.5)  61.5% female | 1.**Empirical referents**   - Functional decline: ≥1 point decrease in ADL performance measured using the MBI (10 items; score range 0-20) - impaired mobility measured by the Timed Up and Go test (TUG)   2. **Observation period**  PM to DC | - Higher premorbid ADL function - Adverse drug reactions - Referral source: in-hospital consultation to geriatrics | - *Decline in ADL performance* - *New or increased assistance required with mobility* | More likely to have a longer length of stay (LOS) |
| 1.Condorhuaman-Alvarado et al.  (translated from Spanish using Deep L)  2. 2017  3. Spain | 1. Retrospective Cohort Study  2. “*To compare baseline characteristics and those found during hospitalization as predictors of functional loss at discharge in elderly patients hospitalized for acute illness” pg 253* | 1.N = 2,557 computerised records of patients admitted to an acute geriatric unit of a tertiary hospital over a 10-year period.  2. Mean age 86.1 ± 6.3  61% female | 1.**Empirical referents**  Functional decline: ≥ 5-point decrease in ADL performance measured using the Barthel Index (BI) (10 items; score range 0-100)  2. **Observation period**  PM to DC | - Older age - Low premorbid ADL function - Cognitive impairment - Greater number of diagnoses - ADL decline before admission - A longer hospital stay - Higher co-morbidity burden | *Decline in ADL performance* | Not reported (NR) |
| 1.Fimognari et al.  2. 2017  3. Italy | 1. Observational prospective study  2. “*To investigate the potential independent role played by comorbidity, and particularly the severity of diseases at admission, in the functional trajectories around hospitalisation for acute medical diseases in older patients”pg 103* | 1.N=696 elderly individuals admitted to 2 general medical units in three Italian hospitals  2.Mean age 82.7 years  53% female | 1. **Empirical referents**  Functional decline: ≥ 5-point decrease in ADL performance measured using the BI (10 items; score range 0-100)  2. **Observation period**  PM to DC | - ADL decline before admission - A longer hospital stay | *Decline in ADL performance* | NR |
| 1.Gagliardi et al.  2. 2018  3. Italy | 1. Multicentre observational study.  2. “Describe the impact of preadmission, decline on functional trajectories among older hospitalised patients” pg 1363 | 1.N=2,011 older inpatients consecutively admitted to 4 medical wards in 3 large hospitals  2. mean age 84.7 ± 6.3  (≥65 years)  56.1 female | 1.**Empirical referents**  Functional decline: decrease in ADL  performance (Inter-RAI 6 items tool)  2. **Observation period**  PM to DC | - Hearing impairment (PM) - visual impairment (PM) - Weight loss (PM) - Cognitive impairment - ADL decline before admission | *Decline in ADL performance* | Functional decline or improvement during hospitalisation |
| 1. Reichardt et al.  2. 2019  3. Netherlands | 1. Prospective longitudinal cohort study  2. “*To identify trajectories of cognitive-affective depressive symptoms among acutely hospitalised older patients and whether trajectories are related to prognostic baseline factors and three-month outcomes such as functional decline, falls, unplanned readmissions, and mortality* “ pg 66 | 1.N = 398 acutely hospitalised adults in a medical, cardiology and geriatric wards in 6 hospitals  2. Mean age 79.6 ± 6.6  (≥70 years)  49% female | 1.**Empirical referents**  Functional decline: decrease in ADL performance measured using the Katz index (6 items; score range 0-6)  2. **Observation period**  PM to FU  (3 months) | Depressive symptom trajectory (mild, severe) | *Decline in ADL performance* | - Post discharge falls within 3 months - Functional decline within 3 months post discharge |
| 1. Yoo et al.  2. 2019  3. South Korea | 1. Longitudinal observational study  2. “*To investigate the trend in functional changes over time and factors associated with the number of areas showing functional decline in older patients who have been discharged from an acute hospital*” pg 192 | 1.N=156 older adults in a  Tertiary hospital  (50.6% in internal medical ward, 35.3% surgical, 11% other departments)  2. Mean age 73.14 ±5.04  (≥65 years)  48.1% female  62.8% lived with spouse  16% lived alone | 1.**Empirical referents**  Functional decline: decrease in ADL performance (longitudinally survey)  2. **Observation period**  AD to FU  (1 and 3months) | - Older age - No caregiver or having a spouse as the care giver (AD to FU1) - Less than elementary school education (AD to FU3) - Being in the medical department in the hospital versus surgical (predictive at 1 month) - A longer hospital stay (AD to FU3) | *Decline in ADL performance* | NR |
| 1. Chou et al.  2. 2020  3. Taiwan | 1. Retrospective Cohort Study  2. “*To explore the predictive effect of physical restraint, use for adverse hospital outcomes, such as functional decline during hospitalisation, longer length of stay and death*” pg 160 | 1.N = 3460  older adults  admitted consecutively to medical wards in a tertiary hospital  (n=188, 8.3 % with restraints)  2.mean age 77.5 ± 8.4 year  (≥65 years)  39.6% female | 1**.Empirical referents**  Functional decline: ≥ 1 point decrease in ADL performance measured using the Katz Index (6 items; score range 0-6)  2. **Observation period**  PM to DC | Physical restraints  (nasogastric tube foley catheter) | *Decline in ADL performance* | - A longer hospital stay - Increased in hospital mortality |
| 1.van Seben et al.  2. 2020  3.Netherlands | 1. Prospective Cohort Study  2. “*To analyse the longitudinal associations between geriatric syndromes and functional decline, readmission and mortality*” pg 1403 | 1.N = 401 acutely hospitalised patients (≥70 years) in six hospitals (medical, cardiology and geriatric wards)  2. Mean age 79.7 ± 6.7  48.6% female | 1. **Empirical referents**  Functional decline: ≥ 1decrease in ADL performance measured using the Katz index (6 item; score range 0-6)  2. **Observation period**  PM to AD to DC to FU (1, 2 and 3 months) | - Mobility impairment - Malnutrition - Pain - Apathy - Falls risk - Fear of falling - Developing or persist incontinence - Cognitive impairment - Depressive symptoms | *Decline in ADL performance* | - Increased risk of readmission to hospital within 3 months - Mortality (mediated through geriatric syndromes) |
| 1. Hajduk et al.  2. 2020  3. USA | 1. Prospective longitudinal cohort Study (secondary analysis)  2. “*To describe the incidence of functional decline among older people hospitalized for AMI in a nationwide cohort study and to develop a risk model for functional decline using a wide array of predictors to aid identification of patients at risk*” pg 2 | 1.N = 2555 older adults with acute myocardial infarction ≥75 years. Ninety-four academic and community hospitals  (exact wards type not specified)  2. Mean age 81.3 (SD, 4.9) Slightly more than half were women | 1.**Empirical referents**   - Functional decline: >1 point decrease in ADL performance (study specific interview) - reduced mobility (Timed up and Go)   2. **Observation period**  PM to FU  (6 months) | - Older age - Low premorbid ADL function - Decline in mobility prior to admission - Depressive symptoms - A longer hospital stay - Impaired mobility during hospitalisation | - *Decline in ADL performance* - *Reduced mobility* | Persistent loss of functional independence at 6-month follow-up |
| 1. Hartley et al.  2. 2021  3. UK | 1. Prospective repeated-measures cohort study  2. “*To investigate changes in knee extension strength and physical function in older patients during and after the contribution of illness severity, frailty and sedentary activities to changes in knee extension strength*” pg 153 | 1.N = 70 recruited, 62 had ≥ 1 repeated muscle strength measure, 49 had follow-up  2. Mean age 84 years (≥75 years)  Female 43% | 1.**Empirical referents**   - Functional decline: decrease in ADL performance measured using the BI (10 items; score range 0-100) - Reduced knee extension, grip strength and functional mobility   (DeMorton  Mobility index (DEMMI))  2.**Observation period**  PM to AD to DC to FU (4-6 weeks) | - Higher baseline knee-extension strength - Higher frailty on admission - Lower inflammatory burden on admission - Low in hospital mobility (greater sedentary time in hospital) | - *Decline in ADL performance* - *Reduced knee extension strength (AD to DC)* | - Persistent functional decline from admission to 4-6 weeks - Persistent muscle strength impairment post-discharge |
| 1. Tavares, Nunes and Gracio  2. 2021  3. Portugal | 1. Prospective Cohort Study  2. “*To identify the predictors of functional decline in hospitalized individuals age 70 or over, between baseline and discharge, discharge and follow-up, and baseline and three-month follow-up*” pg 2 | 1.N=101 older adults (≥70 years) (medical service, 2 female and 2 male wards)  2.Mean age 82.47 ±6.57 years  (≥70 years)  53.3% female | 1.**Empirical referents**  Functional decline: ≥ 1 point decrease in ADL performance measured using the Katz Index (6 items; score range 0-6)  2.**Observation period**  PM to FU  (3 months) | - Limited social supports/not having a partner (PM) - Cognitive impairment - Hospitalisations in the past year - Mechanical restraint - Lung disease (DC to FU) - Episode of delirium during hospitalisation - ISAR-HP greater or equal to 2 (PM to FU) | *Decline in ADL performance* | Increased likelihood of institutionalisation at FU |
| 1. Chou et al.  2. 2021  3. Taiwan | 1. Retrospective Cohort Study  2. “*To develop a predictive model to identify hospitalised older patients at risk of functional decline*” pg 963 | 1.N = 1698 older adults admitted consecutively to a medical ward in a Tertiary Medical Centre  2. Mean age 75.8 ±7.9 years (table 1)  (≥65 years)  39.6% female | 1**. Empirical referents**  Functional decline: ≥ 1 point decrease in ADL performance  Measured using the Katz Index (6 items; score range 0-6)  2. **Observation period**  PM to DC | - Older age - Hearing impairment - History of falls within one year - Risk of malnutrition - Physical restraint - Admission via the emergency department - Hospital stays greater than 5 days | *Decline in ADL performance* | Return to the emergency department within 1, 3 and 6 months |
| 1.Huang et al.  2. 2021  3. Taiwan | 1. Retrospective Cohort Study  2. “*To investigate the prognostic significance of hospitalization-associated muscle weakness for short-term and long-term functional declines among oldest old hospitalized patients*”pg 1 | 1.N=431 older adults Tertiary Medical Centre assessed within 48 hours (Geriatric Evaluation and Management Unit)  2. Mean age 86 ± 6.2 years  21.5% female | 1. **Empirical referents**  Functional decline: >20 points decrease in ADL performance  measured using the BI (10 items; score range 0-100)  2. **Observation period**  AD to DC to  FU (1 and 6 months) | - Lower body mass index (BMI) - Hospital associated muscle weakness (decline in handgrip strength during hospitalisation - Risk of pressure sores (at 6 months) | *Decline in ADL performance* | Persistent functional decline at 1- and 6-months FU |
| 1. Aarden et al.  2. 2021  3. Netherlands | *1*. A Multicentre Prospective Observational Cohort Study*.*  *2. “To determine the longitudinal changes in muscle mass, handgrip strength, and physical performance from hospital admission up to 3 months post-discharge, adjusting for depressive symptoms, cognitive fatigue, fear of falling, risk of malnutrition, and comorbidity, in acutely hospitalised older adults” pg 840* | 1.N = 343 acutely hospitalized older adults in six hospitals (medical, cardiology and geriatric wards)  2. mean age 79.3 ± 6.6 years  (≥70 years)  49.3% female | 1.**Empirical referents**  Functional decline: observable change in muscle mass, muscle strength, chair stand and gait speed measured using the short physical performance battery (SPPB), dynamometer and bio-electrical impendance analysis (BIA))  2. **Observation period**  AD to DC to FU  (3 months) | NR | Muscle loss and reduced physical performance during hospitalisation | - Decrease in muscle mass - Significant improvement in chair stand performance - Significant improvement in gait speed |
| 1. Tavares et al.  2. 2021  3. Portugal | 1. A secondary analysis of data from a Prospective Cohort Study  2*. “To examine if frailty, measured with the Frailty Index (FI), can predict functional decline during and after hospitalisation*” pg 2 | 1. N = 101 older adults at a  University hospital (4 internal medical wards)  57.4% were frail  2. Mean age 82.47 ± 6.57  (≥ 70 years)  53.3% female | 1.**Empirical referents**  Functional decline: ≥ 1 point decrease in ADL performance measured by the Katz index (6 items; score range 0-6)  2.**Observation period**  PM to FU  (6 months) | - Older age - Frailty - Not being married (PM to DC only) | *Decline in ADL performance* | Persistent functional decline at 3 and 6 months post-discharge |
| 1. Kolk et al.  2. 2021  3. Netherlands | 1. Prospective Observational Cohort Study  2. (i) “*To determine the number of steps taken by older patients in hospital and 1 week after discharge: to identify factors associated with step numbers after discharge; and to examine the association between functional decline and step numbers after discharge”* pg 425 | 1.N =188 older adults acutely admitted to six hospitals (medical, cardiac and geriatrics wards)  2. Mean age 79.1 ± 6.7 years  (≥70 years)  43.6% female | 1.**Empirical referents**  Functional decline: ≥1 point decrease in ADL performance measured using the Katz index (6 items; score range 0-6)  2.**Observation period**  PM to DC to FU  (1 months) | NR | *Decline in ADL performance* | Persistent functional disability at 1 months |
| 1. Bielza et al.  2. 2022  3. Spain | 1. Prospective Cohort study  2. “*To investigate the feasibility and accuracy of four frailty instruments: FRAIL, Clinical Frailty Scale (CFS), hand grip strength and the Spanish Frailty-VIG; and to evaluate their ability to predict adverse outcomes in an acute care setting*” pg 827 | 1. N =185 older adults consecutively admitted to an Academic Tertiary Care Hospital  (geriatric medicine)  2. Mean age 89 years (85.0-93.0)  60.5% female  (≥ 70 years)  63.9% were living with a dementia | 1.**Empirical referents**  Functional decline: ≥ 5-point decrease in ADL performance measured by the BI  (10 items; score range 0-100)  2.**Observation period**  PM to DC to FU  (3 months) | Frailty at admission (as defined by frailty instruments) | *Decline in ADL performance* | Persistent functional decline at 3 months |
| 1. Le Gentil et al.  2. 2022  3. France | 1. Case Control Study  *2. “To determine whether COVID-19 per se is an independent prognostic factor for functional decline beyond advanced age, associated comorbidities and disease severity” pg 896* | 1.N =264 older adults consecutively admitted to a University Hospital with a diagnosis of Covid 19  (acute geriatric ward)  2. Mean age 87  (≥75 years)  61% female | 1.**Empirical referents**  Functional decline: ≥ 1point decrease in ADL performance measured by the Katz index  (6 items; score range 0-6)  2. **Observation period**  PM to FU (3 months) | - A history of falls - Malnutrition - Low premorbid ADL function - A longer hospital stay - Co-morbidities | *Decline in ADL performance* | NR |
| 1. Nagae et al.  2. 2022  3. Japan | 1. A Prospective Observational Cohort Study  2. “*To examine the association of muscle evaluation, including muscle ultrasound, with hospital-associated disability, focusing on ADL categories”* pg 681 | 1.N: 256 older adults admitted to a geriatric ward of an acute hospital  2. mean age 85.2±5.9  (≥65 years)  58.2% female | 1.**Empirical referents**  Functional decline: ≥10% decrease in ADL performance measured by the  BI (10 items; score 0-100)    2. **Observation period**  PM to DC | Reduced bilateral thigh muscle thickness (BATT) | - *Reduced mobility* - *Decline in ADL performance* | NR |
| 1. Freeman et al.  2. 2023  3. USA | 1. Retrospective cross-sectional analysis  *2. “To examine the impact of delirium during hospitalisation on HAD across a wide spectrum of hospital units using a standardised delirium assessment tool” pg 534* | 1.N = 33,111  (18,775 medical patients admitted to an acute hospital) comparing patients with and without delirium related to HAD  2. (≥ 65 years)  Gender not specified | 1.**Empirical referents**  Functional decline: decreased performance in ADLs measured by the Katz index (6 items; score range 0-6)  2. **Observation period**  AD to DC | - Older age - Co-morbidity score - Admission ADL function - Cognitive impairment - Delirium - LOS ≥ 8 days | *Decline in ADL performance* | NR |
| 1. Nagae et al.  2. 2023  3. Japan | 1. Prospective Cohort Study  2. “*To examine whether dehydration is a predictor of hospital-associated disability categorized into mobility and self-care impairment among acute hospitalized older adults*”pg 113 | 1.N =192 older adults admitted to a geriatric ward in an acute hospital  2. mean age 84.7 ±5.8 years  (≥65 years)  58.9% female | 1. **Empirical referents**  Functional decline: ≥10% decrease in ADL performance measured using the BI (10 items; score range 0-100)  2.**Observation period**  PM to DC to FU  (3 months) | Dehydration at admission | - *Decline in ADL performance* - *Decline in mobility* | Persistent functional decline at 3 months post DC |
| 1. Chenhuichen et al.  (Article translated from Spanish using Deep L)  2.2024  3.Spain | 1. Observational retrospective cohort study  2. “To analyse the epidemiological, clinical and functional characteristics of patients admitted to the University Hospital of Navarra due to SARS-CoV-2 infection, as well as the predictors of mortality, during the first wave of the pandemic caused by the virus” pg 1 | 1.N= 426 older patients who tested positive for SARS-CoV-2 in an Acute Hospital  2. Mean age 83.2  (≥ 75 years)  47.4% female | 1.**Empirical referents**  Functional decline: ≥ 10 point decrease performance in ADLs measured by the BI (10 items; score range 0-100)  2. **Observation period**  PM baseline to DC and FU (1 month) | - Immobility at discharge - Pressure sores present on admission - Incontinence on admission - Falls in hospital | - *Decline in ADL performance* - *Reduced mobility* | Persistent functional decline at 1 month FU |
| 1. Clotet-Vidal et al.  2. 2024  3. Spain | 1. Prospective Observational Study  2. “*To analyse functional decline, institutionalisation, malnutrition, and risk factors after hospital admission for Community Acquired Pneumonia* (*CAP)” pg 1* | 1.N = 144 older adults with a Community Acquired Pneumonia (CAP) admitted to a Tertiary University Hospital  2. Mean age 77.15±7.91 years  (≥65 years)  44.4% female | 1.**Empirical referents**  Functional decline: ≥10-point decrease in ADL performance measured by the BI  (10 items; score range 0-100)  2.**Observation period**  Premorbid to FU (30-60 days) | - Older age - Low premorbid ADL function - Co-morbidity burden - Previous institutionalisation - A longer hospital stay | *Decline in ADL performance* | Institutionalisation |
| 1. Gatenio-Hefling et al.  2. 2025  3. Israel | 1. Observational longitudinal study  2. “*To examine the contribution of the Timed Up and Go (TUG) test in predicting Hospitalisation Associated Functional Decline (HAFD) among older adults, able to independently ambulate before admission” pg 1* | 1.N = 310 older adults admitted to one of  two acute medical wards  2. Mean age 77±7  (≥65 years)  47.10% female | 1.**Empirical referents**  Functional decline: decrease in ADL performance measured using the BI (10 items; score range 0-100)  2. **Observation period**  PM to DC | Impaired baseline mobility  (TUG ≥ 12 seconds or inability to complete TUG on admission) | *Decline in ADL performance* | Functional  D decline  at discharge |
| 1. Morioka et al.  2. 2025  3. Japan | 1.Retrospective cohort study  2.” *To investigate the relationship between nurse staffing shortfalls during typical day and night shifts and the risk of HAD in older adults in acute care hospitals”pg 1* | 1.N=30,019 medical patients admitted to nine acute care hospitals  2. Mean age 76  (≥65 years)  41.7% female | **1. Empirical referents**  Functional decline: observable functional dependency    (Japanese Severity of a Patient’s Condition and Extent of a Patient’s Need for Medical/Nursing Care Scale)  **2. Observation period**  AD to DC | Lower nursing staff levels | *Decline in ADL performance* | Functional decline at discharge |

Footnote: Only outcomes statistically significant in multivariable analyses are reported. Empirical referents are defined as observable clinical indicators used to operationalise hospital-associated decline; measurement tools are reported where relevant. Observation periods are coded as premorbid (PM): self-reported baseline function prior to admission (as recorded on admission); Admission (AD): functional status measured during hospital admission; Discharge (DC): functional status at hospital discharge; Follow-up (FU): post-discharge assessment as defined by each study.

Abbreviations; **NR**, Not reported; **AD,** Admission; **ADL**, Activities of Daily Living**; BIA,** Bio-electrical impendance Analysis; **DC,** discharge**; DEMMI**, de Morton Mobility Index; **FU,** follow-up; **iADL**, Instrumental Activities of Daily Living; **ISAR**, Identification of Seniors at Risk; **interRAI,** International Resident Assessment Instrument; **OARS**, Older Americans Resources and Services Questionnaire; **TUG**, Timed Up and Go**; SPPB**, Short Physical Performance Battery; FIM, Functional Independence Measure; **CGA**, Comprehensive Geriatric Assessment**; AGU**, Acute Geriatric Unit; **CAP**, Community Acquired Pneumonia; **PM,** Premorbid; **BI,** Barthel Index**; BMI,** Body Mass Index; **BATT**, Bilateral Anterior Thigh Thickness; **HAD**, Hospital Associated Decline; **SR**, self-reported
