# Supplementary material for: Hospital-associated decline: A concept analysis
Source: Int J Nurs Stud Adv. 2026 Oct 3;11:100700. doi: 10.1016/j.ijnsa.2026.100700 (PMC13683459; doi:10.1016/j.ijnsa.2026.100700)
Supplement: Supplementary file 5 [file mmc5.docx]

**Supplementary Material File S4. Model, Contrary and Related Cases, with consideration of a Borderline Case of Hospital-associated Decline**

**S4.a Model Case of Hospital-associated Decline**

The model case illustrates hospital-associated decline as a complex, time-defined process occurring in the context of patient vulnerability, acute illness, and hospital care processes, manifested by a measurable decline in activities of daily living performance.

Mrs O’Connor is an 87-year-old widow who lived independently prior to admission, managing all activities of daily living, including dressing, bathing, toileting, feeding, and mobility, without assistance. Her daughter assisted with shopping, cooking and managing finances. Although functionally independent, she had pre-existing frailty and cognitive impairment, indicating increased vulnerability to decline during hospitalisation *(Antecedent: Patient vulnerability).*

Mrs O’Connor was admitted to a medical ward with a respiratory tract infection (*Antecedent: acute illness).* On admission, her frailty and increased care needs, including assistance with mobility, toileting and personal care, were recognised by nursing staff. She was allocated to a room close to the nursing station, with greater availability of nursing and healthcare-assistant support, enabling closer observation and assistance with care. This represented an adaptive nursing response to her identified vulnerability and care complexity.

During the initial days of hospitalisation, her acute illness improved with medical treatment. However, her mobility remained substantially reduced. Her pre-existing frailty and cognitive impairment indicated ongoing vulnerability during recovery. She spent prolonged periods in bed, with limited opportunities and encouragement to mobilise *(Antecedent: hospital care processes, patient vulnerability and acute illness).*

Over the course of the admission, these antecedent domains were present alongside progressive deterioration in Mrs. O’Connor’s physical function. She developed new dependence in dressing, toileting, and transferring from bed to chair *(Attribute: decline in activities of daily living performance*).

By discharge, despite improvement in the acute respiratory illness, Mrs. O’Connor required assistance with several self-care tasks and used a walking frame to mobilise short distances. Her functional status was therefore lower than before she became acutely unwell. New social supports were therefore required to facilitate discharge home (*Consequence: new care needs following discharge).*

**Reflection**

This case illustrates hospital-associated decline as a time-defined process in which patient vulnerability, acute illness, and hospital care processes may co-occur across the hospitalisation episode alongside a measurable decline in activities of daily living performance. Informed by nursing complexity literature, the case extends beyond the empirical findings to illustrate how the recognition of patient vulnerability and increasing care complexity may prompt an adaptive nursing response. The occurrence of hospital-associated decline despite an adaptive nursing response further illustrates the complexity of the concept and the potential for multiple antecedent domains to co-occur across the hospitalisation episode. Despite improvement in her acute illness, her activities of daily living performance declined from the level maintained before becoming acutely unwell, resulting in new care needs at discharge. Comparison of function before acute illness and at discharge provides an empirical means of identifying this decline.

**S4.b Borderline Case**

In Walker and Avant’s (2019) framework, a borderline case is typically one that exhibits most, but not all, of the defining attributes of a concept, illustrating a situation where the concept is partially but not fully present. However, in this analysis, no borderline cases of hospital-associated decline were identified. Reduced performance in activities of daily living was identified as the sole defining attribute of hospital-associated decline. As a borderline case exhibits some, but not all, of the defining attributes, a meaningful borderline case could not be constructed when only one defining attribute was identified.

**S4.c Contrary Case**

In Walker and Avant’s (2019) framework, a contrary case is an example that clearly does not represent the concept. It does not contain the defining attributes of hospital-associated decline and is used to illustrate what is not hospital-associated decline.

Mrs Walsh, an 85-year-old retired teacher, was admitted to the hospital with a urinary tract infection *(Antecedent: acute illness).*

Before admission, she lived independently, although she had mild frailty and used a walking stick outdoors (*Antecedent: patient vulnerability).*

On admission, she was mildly unwell but remained mobile with the aid of her walking stick *(Baseline: activities of daily living performance maintained).*

The nursing staff were aware that she was normally independent and, as part of their care plan they encouraged her to mobilise regularly to the bathroom and to wash and dress independently.

A Physiotherapy reviewed her early in her stay and provided short walking exercises, which she completed.

She recovered from her infection and maintained her baseline function and mobility.  She returned home at the same functional level as before admission.

**Reflection**

This contrary case demonstrates the absence of the defining attribute of hospital-associated decline. Despite patient vulnerability, acute illness, and hospitalisation, Mrs Walsh maintained her premorbid functional status across the admission-discharge period and did not develop new dependence in activities of daily living. This case demonstrates that the presence of antecedent domains does not constitute hospital-associated decline. Measurable decline in activities of daily living performance is required, thereby helping to clarify the boundaries of the concept. The case also provides an example, informed by nursing complexity literature, of how recognition of premorbid function and care needs can inform an adaptive nursing response intended to support mobility and independence across the hospitalisation episode.

**S4.d Related Case**

In Walker and Avant’s (2019) concept analysis framework, a related case is used to show a situation that resembles the concept but is not the same as the concept.

Mr Lynch, an 85-year-old man with advanced dementia and frailty, was admitted to hospital with dehydration following a viral illness (*Antecedent: acute illness).*

Before admission, he required assistance with activities of daily living and a hoist to transfer from bed to chair*. (Antecedent: patient vulnerability: pre-existing functional dependency and frailty).*

During his hospital stay, his care needs remained unchanged, he continued to need assistance with all activities of daily living, consistent with his baseline functional status.

There was no measurable change in his level of independence compared with before admission.

**Reflection**

This related case illustrates pre-existing functional dependency, a related but distinct concept. It resembles hospital-associated decline because both involve limitations in activities of daily living but does not meet the defining attribute because Mr Lynch’s level of dependency did not deteriorate during the hospitalisation episode. Although he was hospitalised with an acute illness, his level of functional dependence remained stable from before admission to discharge. The case therefore distinguishes hospital-associated decline – a demonstrable deterioration in activities of daily living performance associated with the hospital episode – from pre-existing or progressive functional impairment.
